# Phase Coexistence in Hamiltonian Hybrid Particle–Field Theory Using a Multi-Gaussian Approach

**DOI:** 10.1021/acs.jpcb.4c05525

**Published:** 2024-11-14

**Authors:** Samiran Sen, Henrique Musseli Cezar, Morten Ledum, Xinmeng Li, Michele Cascella

**Affiliations:** Hylleraas Centre for Quantum Molecular Sciences and Department of Chemistry, University of Oslo, P.O. Box 1033 Blindern, Oslo 0315, Norway

## Abstract

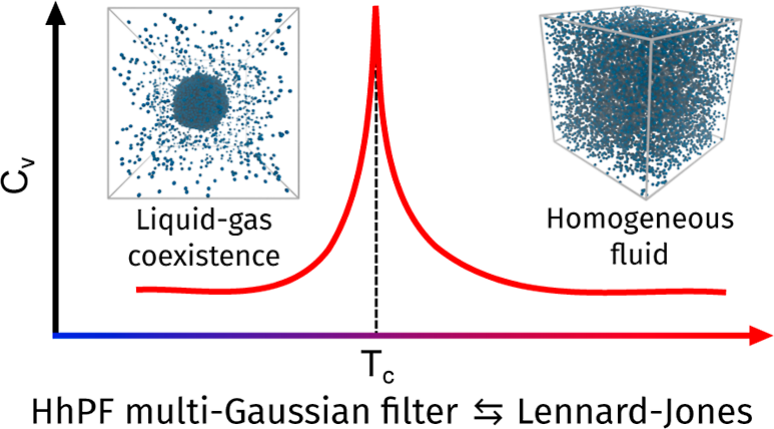

This study introduces an implementation of multiple Gaussian filters within the Hamiltonian hybrid particle-field (HhPF) theory, aimed at capturing phase coexistence phenomena in mesoscopic molecular simulations. By employing a linear combination of two Gaussians, we demonstrate that HhPF can generate potentials with attractive and steric components analogous to Lennard–Jones (LJ) potentials, which are crucial for modeling phase coexistence. We compare the performance of this method with the multi-Gaussian core model (MGCM) in simulating liquid–gas coexistence for a single-component system across various densities and temperatures. Our results show that HhPF effectively captures detailed information on phase coexistence and interfacial phenomena, including microconfiguration transitions and increased interfacial fluctuations at higher temperatures. Notably, the phase boundaries obtained from HhPF simulations align more closely with those of LJ systems compared to the MGCM results. This work advances the hybrid particle-field methodology to address phase coexistence without requiring modifications to the equation of state or introducing additional interaction energy functional terms, offering a promising approach for mesoscale molecular simulations of complex systems.

## Introduction

**Figure 1:**
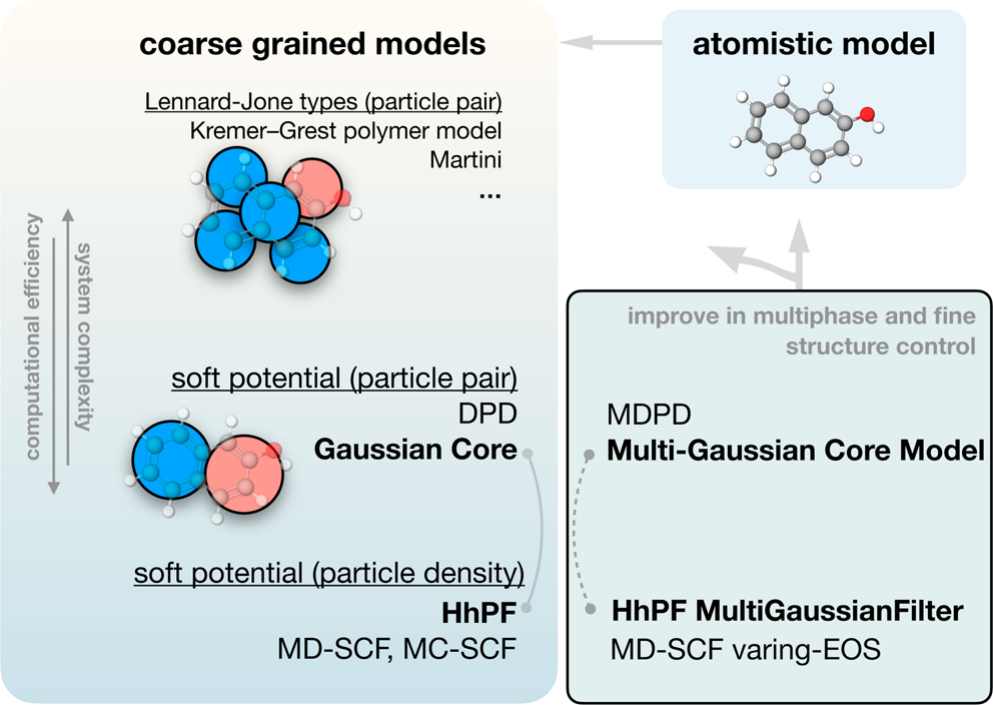
Overview of different types of coarse-grained models in molecular simulation categorized by their nonbonded interaction potential forms.

Mesoscopic molecular simulation methods bridge the gap between atomistic simulations and macroscopic continuum models by grouping atoms into computationally manageable units known as coarse-grained beads. By reducing the number of degrees of freedom, coarse-graining enables the simulation of larger systems over longer time scales compared to fully atomistic models. The primary distinction between different mesoscopic models lies in their descriptions of interactions between coarse-grained beads (Figure 1), leading to variations in both accuracy and computational efficiency.

The most straightforward approach involves the application of two-body interaction potentials such as Lennard-Jones (LJ) in coarse-grained models. Models employing this strategy include, for example, the Kremer–Grest (KG) polymer model^1^ and the Martini model^2,3^ for polymer physics and biological systems, respectively. Despite their strengths in handling complex systems, these models face significant computational demands compared to other mesoscopic alternatives. The LJ potential used in these models, which includes a strong repulsive component (e.g., 1/*r*^12^), restricts the time steps to maintain numerical stability, and the more intricate potential energy surface compared to other methods may lead to kinetic trapping.

Other mesoscopic modeling approaches employ much simpler potentials, more suitable for highly coarse-grained levels, in the form of single repulsive potentials. These potentials have roots in Flory–Huggins theory and self-consistent field theory^4−6^ in polymer physics. A prominent technique in this domain is dissipative particle dynamics (DPD).^7^ DPD is a coarse-grained method that represents groups of atoms as single particles interacting through soft quadratic potentials, efficiently capturing hydrodynamic behavior.^8^ Another approach involves the Gaussian potential or the Gaussian core model (GCM), first introduced by Stillinger^9^ in the study of fluid and solid phases. Compared with DPD, GCM introduces a dimension for the bead size, enhancing its versatility and applicability. In recent years, mesh-based density methods have emerged. Although the particles interact via density functionals in these methodologies, they essentially adopt soft-core potentials. For example, the MC-SCF^10^ and MD-SCF^11,12^ methods mimic the quadratic potentials in DPD, while the Hamiltonian hybrid particle-field (HhPF) method, which employs Gaussian filters within a mesh, resembles the Gaussian core model.^13,14^

Soft potentials generally improve phase space sampling efficiency by simplifying and smoothing energy landscapes, but efficiency may still be limited in some cases. For instance, soft interactions can cause systems to get stuck in a local configuration or enter into a state that resembles a glass transition due to kinetic slowdown and reduced mobility, especially at low temperatures and high densities.^15−17^

To observe phase coexistence in a model, both attractive and repulsive components must be present in the microscopic interactions. Existing implementations, which only carry the repulsion, are suitable for dense and homogeneous systems, but cannot handle liquid–gas coexistence and lose fine-structure control. Recent progress has been made to address these limitations. The many-body DPD (MDPD) method introduces a density-dependent attractive term into a conservative force, enabling the formation of vapor–liquid coexistence.^18,19^ For the MD-SCF, recent work introduces new equations of state (EOS). Sevink et al. proposed a modified Hamiltonian for the hybrid particle-field (hPF) method that replaces the usual compressibility term with terms that include higher-order density contributions in the EOS.^12^ This change in the Hamiltonian is argued to be necessary to achieve phase coexistence. Another inspiring development comes from the Gaussian core side. The Multi-Gaussian Core Model (MGCM) can introduce both positive and attractive components, enabling the study of interface problems and complex systems. They can also approximate targeting potentials, which is favorable in machine learning algorithms.^20,21^

Recently, we demonstrated how the HhPF is in principle equivalent to any two-body potential, depending only on the choice of the filter function.^14,22^ Continuing on this development line, this paper implements multiple Gaussian filters to reproduce the improvements accessible to the MGCM method, showing that it is possible to capture the essence of multiphase fluid behavior by utilizing only an optimal choice of the filter function.

## Methods

This section reviews the fundamental principles underlying the MGCM and HhPF theories, subsequently progressing to the derivation of the Multi-Gaussian filters within the HhPF framework.

### Multi-Gaussian Core Model

In the GCM,^9,23^ the interaction potential between two particles positioned at **r**_*k*_ and **r**_*l*_ is given by
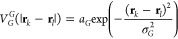
1where σ_*G*_ denotes the extent of interaction and is in distance units and *a*_*G*_ is an energy scale.

For the MGCM, we write the interaction potential as a linear combination of *N*_*G*_ potentials of the GCM model. For a system of *N* particles
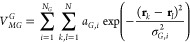
2where the linear combinatorial coefficients are absorbed into the energy-scales *a*_*G*,*i*_. We note that the usual negative sign preceding the *a*_*G*_ parameters has been omitted in alignment with the Gaussian functions utilized in our HhPF methodology. As a result, a positive value of *a*_*G*_ implies a repulsive potential, and conversely, a negative value denotes an attractive potential.

### HhPF Theory

Within the HhPF framework, the particle density ϕ(**r**) is given by
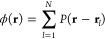
3where *P* is a window function that distributes the number densities associated with particle *l*, located at **r**_*l*_, in space, and which has a total of *N* units. ϕ(**r**) is then filtered via a convolution with a *filter function*
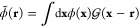
4

Nonbonded interaction energies are defined in terms of particle densities through an energy functional , while bonded (intramolecular) energies are defined through a standard particle–particle energy term . A system of *M* molecules, with the *i*th molecule having *N*_*i*_ particles is then subjected to the Hamiltonian
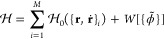
5
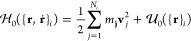
6where  is the Hamiltonian for the *i*th molecule, comprising the bonded potential energy of a molecule , and the kinetic energy , where, **v**_*j*_ is the velocity of the *j*th particle in the *i*th molecule. The functional of interaction energy  is the usual Flory–Huggins mixing term plus the incompressibility term that is usually employed in HhPF.^13^ In terms of a local energy density functional , this functional can be written as
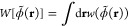
7

The force due to the field interaction on a particle placed at **r**_*i*_, can be obtained by direct differentiation of the interaction energy functional  as
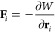
8

Since the functional dependence of *W* on the filtered particle densities  involves a convolution (see 4), in practice, we perform the force calculations numerically in reciprocal space with fast Fourier transform (FFT) operations. Further details can be found in refs (13, 22, 24).

### HhPF Filter Formalism from MGCM Potential

We choose the density filtering function , introduced in the previous section, to be a weighted sum of *N*_*G*_ Gaussians *g*_*i*_’s with real coefficients *a*_*i*_ and spreads σ_*Hi*_ as
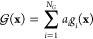
9
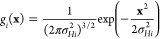
10

According to (4), the filtered density ϕ̃(**x**) is then
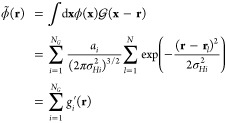
11

We consider a simple interaction energy of the form
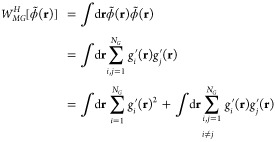
12

The first term in the first integral of (12) yields
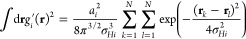
13

To carry out the integral in the second term of (12), we make use of the generalized Gaussian product-rule.^25^ For each cross-term in the sum, we obtain
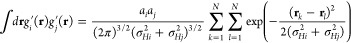
14

We can cast this cross term in the same form as that of the self-term in (13) as
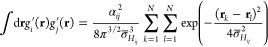
15where
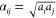
16
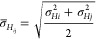
17

We note that α_*ij*_ is the geometric mean of *a*_*i*_ and *a*_*j*_, and  is the arithmetic mean of *σ*_*Hi*_^2^ and *σ*_*Hi*_^2^. The detailed derivation of eqs 15–17 is provided in the Supporting Information. Then
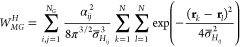
18where,  is simply (*a*_*i*_, σ_*Hi*_) written in matrix notation. We highlight that only *i* = *j* terms are independent constants, while *i* ≠ *j* terms are determined from them.

An HhPF filter comprising *N*_*G*_ Gaussians (9) leads to an energy expression comprising *N*_*G*_ (*N*_*G*_ + 1)/2 distinct terms (18). Because of this, unlike in the case of GCM with a single Gaussian,^14^ there is no simple one-to-one correspondence between the Gaussian potential parameters *a* and σ in HhPF and MGCM.

### Simulation Setup for Liquid–Gas Coexistence

We conducted the simulations in the *NVT* ensemble, using a system of monatomic particles (*N* = 8788) uniformly distributed in cubic boxes of varying side lengths to achieve different densities, across a range of temperatures. This approach allows us to observe phase formation from far-from-equilibrium states, focusing particularly on liquid–gas coexistence. Although a more precise phase boundary determination could be achieved using more refined techniques such as Histogram Reweighting Monte Carlo,^26^ Transition Matrix Monte Carlo,^27^ and Expanded Wang–Landau simulations,^28^ our primary goal was to compare coexistence phases derived from two-Gaussian filter HhPF simulations with those obtained from MGCM methods.

HhPF simulations use SI units of measurement. To increase generality, the MGCM simulations as well as the comparative analysis between the different phases obtained in HhPF and MGCM were performed by adopting standard reduced units. The conversion between the used units are provided in Supporting Information. Quantities reported in reduced units are marked with an asterisk (*).

A comprehensive list of all simulated systems and a summary of the observed phases are provided in Tables S1 (HhPF) and S2 (MGCM) in the Supporting Information. Simulations using both HhPF and MGCM for all were performed using the parameters of Table 1.

**Table 1:** Fitted Parameters for the MGCM and HhPF Interaction Potentials

potential	*a*_*G*1_	*a*_*G*2_	σ_*G*1_	σ_*G*2_
*V*_2*G*_^*G*^	[kJ mol^–1^]		[nm]	
	29.288	–8.291	0.208	0.304

potential	α_*H*1_	α_*H*2_	σ_*H*1_	σ_*H*2_
*W*_2*G*_^*H*^	[kJ mol^–1^]		[nm]	
	1.151	–0.689	0.10	0.22

For the HhPF simulations, we used the HyMD software.^22,29^ Each system underwent a 5 ns equilibration period before data collection, sufficient for phase formation due to the enhanced sampling capabilities of our HhPF methodology.^22^ We determined an optimal time step of 200 fs after extensive testing (4–300 fs range), ensuring the time step does not interfere with phase-formation processes. The size of the mesh grid r* was set to 0.117, which corresponds to 0.04 nm in molecular dynamics units. The temperature was maintained using the canonical velocity rescaling thermostat^30^ with a coupling constant of 0.1 ps.

MGCM simulations were performed using the LAMMPS software.^31^ Each simulation was run for at least 10 ns before data collection, using a 4 fs time step. Temperature control was implemented using the default Nosé–Hoover thermostat^32,33^ with a damping interval of 400 fs and a chain length of 3 thermostats.

## Results and Discussion

### Implementation of Two-Gaussian Filters

In this study, as a proof of concept, we have chosen to employ two Gaussians (*N*_*G*_ = 2) for both the MGCM and the HhPF methodologies. These potentials, comprising both a repulsive and an attractive component, constitute a minimalistic model to capture the liquid–gas coexistence phenomena. In the following, *V*_2*G*_^*G*^ will denote the MGCM potential and *W*_2*G*_^*H*^ the HhPF energy functional.

**Figure 2:**
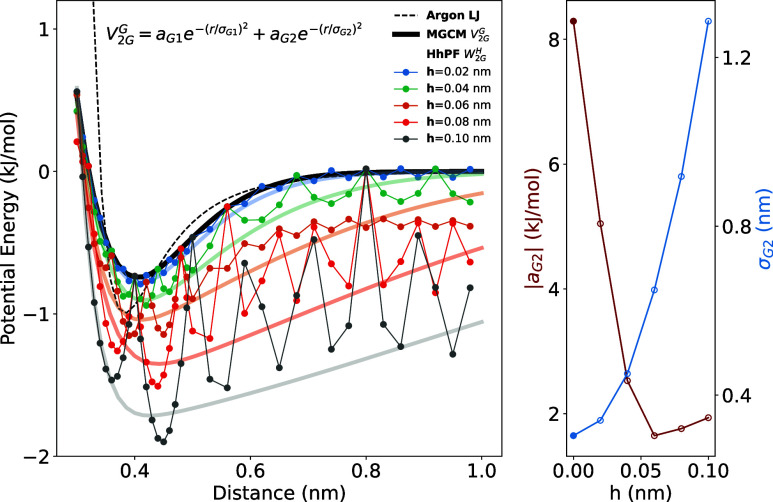
(Left) Potential energy curves *W*_2*G*_^*H*^ derived from HhPF simulations of two beads at varying distances using different grid sizes *h*, shown as color-coded scatter plots. These HhPF potentials are refitted to two-Gaussian potentials *V*_2*G*_^*G*^, depicted by translucent color lines. The targeted two-Gaussian potential and the argon LJ potential are also shown for comparison. (Right) Weight |*a*_*G*2_| and width σ_*G*2_ parameters of the attractive component in the refitted potentials for each *h* case. *h* = 0 denotes the target potential in MGCM and an HhPF simulation with an infinitely dense mesh.

Given the widely studied phase behavior of argon,^34^ we initially selected the Gaussian parameters {*a*_*Gi*_, σ_*Gi*_} for the MGCM *V*_2*G*_^*G*^ of (2) to closely emulate the LJ potential for this element. Subsequently, we derived the Gaussian filter parameters {α_*i*_, σ_*Hi*_} for the HhPF *W*_2*G*_^*H*^ of (18) using numerical fitting. The parameters are given in Table 1. Although the selected potential *W*_2*G*_^*H*^ presents an attractive well that is shallower and wider than the one in the argon LJ potential, shown in Figure 2, it still enables the study of gas–liquid coexistence. We note that by employing a stronger and deeper positive Gaussian component, a closer approximation to the LJ potential can be achieved, particularly in the 0.3 to 1 nm range. However, incorporating such a positive component requires a dense mesh in HhPF, resulting in a high computational cost (Figure S1). For this reason, simulations exploring this set of parameters were not pursued in the present study.

Analysis of *W*_2*G*_^*H*^ potentials under various grid sizes *h* (Figure 2) reveals deviations from the target potential *V*_2*G*_^*G*^ due to the effect of the grid. When bead positions align precisely with the grid, the potential closely replicates the target potential, mimicking an infinitely dense grid scenario. However, as positions deviate from the grid, the potential weakens and the central grid positions experience the greatest disparity. This effect is amplified by larger grid sizes. In particular, *h* = 0.02 exhibits a minimal grid effect. To balance computational efficiency and accuracy, we chose a grid size of *h* = 0.04 for all HhPF simulations. Note that this value is exceptionally small compared to the typical values used for hPF simulations. This small value is needed because of the narrow repulsive Gaussian filter σ_*H*1_ = 0.1 nm. This aligns with our empirical observation that the optimal grid size is typically slightly less than half of the smallest characteristic distance in the simulated system. This finding highlights a potential limitation of the HhPF methodology: its challenge in accurately reproducing smaller spatial scales while maintaining computational efficiency. Future research should focus on addressing this trade-off to enhance the method’s applicability to a broader range of systems.

A well-established principle in particle-mesh methods is that potentials derived from indirect computations through density projections onto numerical grids tend to be more diffuse than those calculated directly from pairwise distances.^35,36^ Our study provides further evidence for this phenomenon. By fitting the *W*_2*G*_^*H*^ potentials to a two-Gaussian model and analyzing their attractive components, we observe a clear trend: as the grid size *h* increases, the attractive component becomes wider and less pronounced. This effect is quantitatively demonstrated by the decreasing weight parameter |*a*_*G*2_| and the increasing width parameter σ_*G*2_ in Figure 2. These findings highlight the importance of carefully considering grid resolution in particle-mesh methods, as it can significantly influence the effective interactions between particles, and consequently the overall system behavior.

### Liquid–Gas Coexistence

The traditional van der Waals theory provides a mean-field approximation of phase behavior for liquid–gas coexistence in the thermodynamic limit as *N* → *∞* and *V* → *∞*, while maintaining a constant density . Beyond the mean-field approximation, realistic finite systems exhibit richer microscopic details due to interfacial effects.^37^ Below the critical temperature, the liquid–gas coexistence phase exhibits intricate microscopic structures, such as spherical droplets or cylindrical liquid regions.

**Figure 3:**
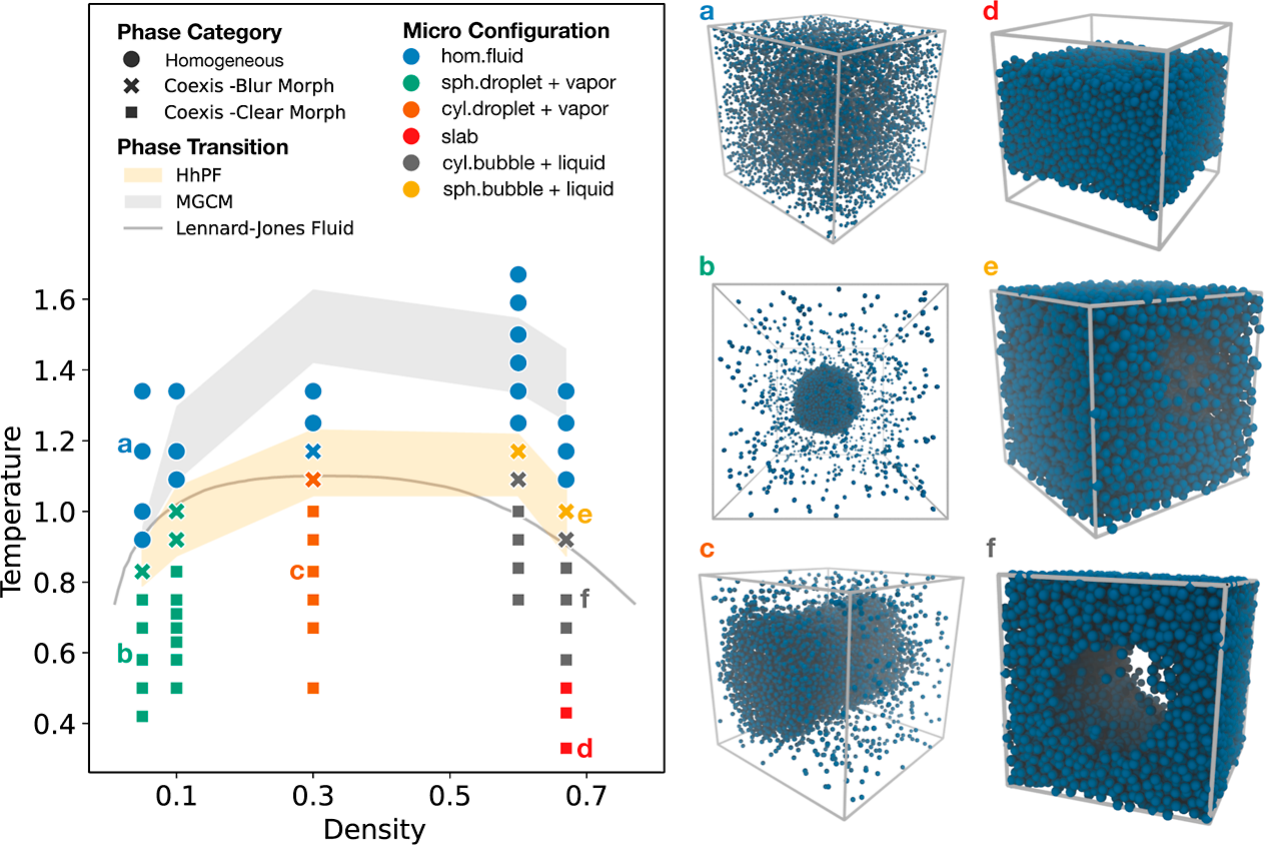
Liquid–gas phase diagram from HhPF simulations of single bead systems. Simulations were conducted at various temperatures along five different densities. Each simulated case is represented by a point, with shape indicating the phase category and color showing the detailed microconfiguration. Snapshots illustrate different microconfigurations on the right side. The shaded zones correspond to the transition region between homogeneous and coexistence phases. The gray zone represents the results of the MGCM simulations. The gray curve shows the phase boundary from MD simulations of LJ systems by Watanabe et al.^38^

**Figure 4:**
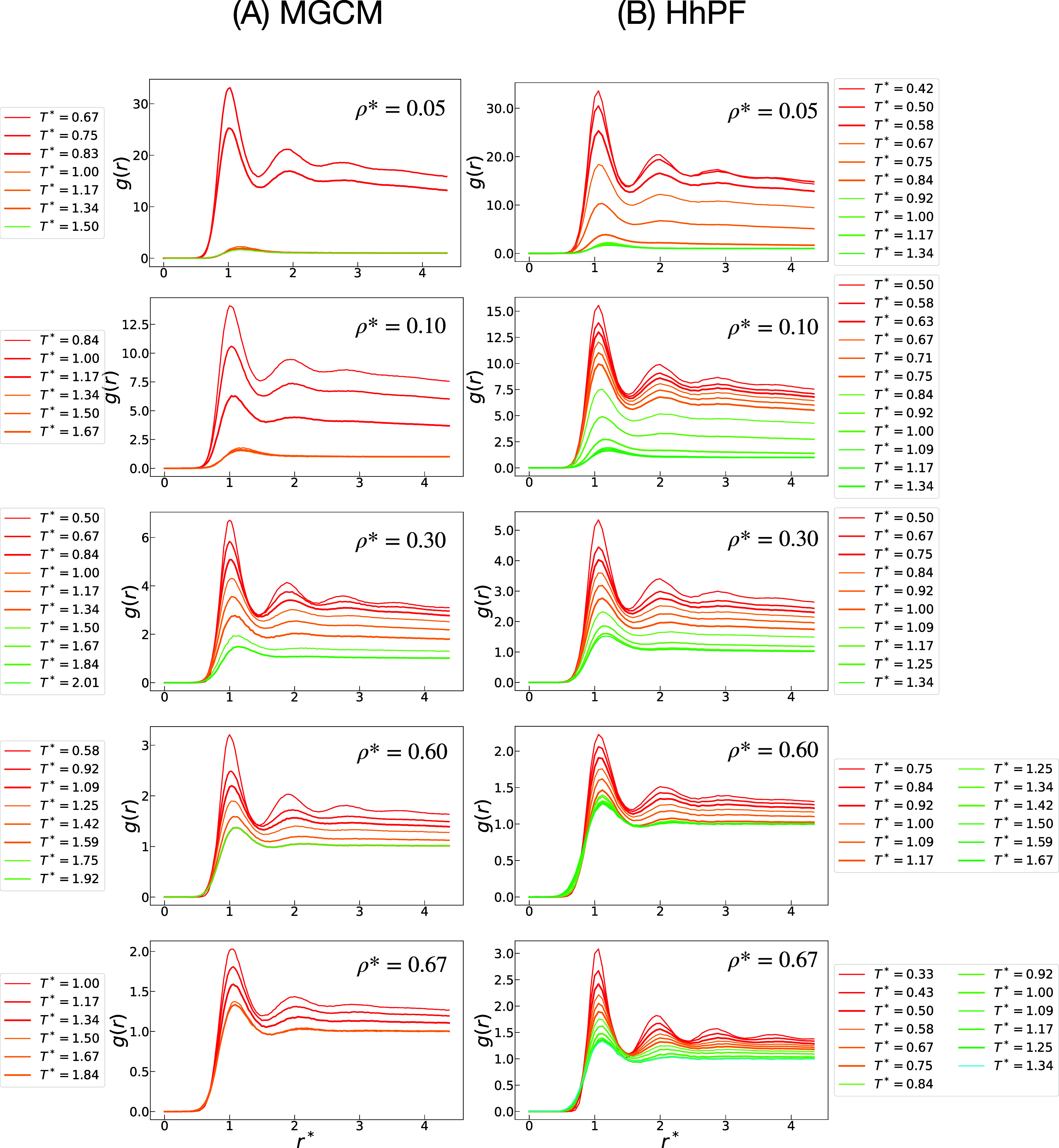
RDFs *g*(*r*) obtained from the MGCM simulations, shown in (A), and HhPF simulations, shown in (B), of systems across different densities ρ* and temperatures T*. All quantities are in reduced units.

Figure 3 illustrates the phase diagram results from HhPF simulations at reduced densities ρ* = 0.05, 0.1, 0.3, 0.6, and 0.67. Above the critical temperature for each ρ*, the systems transition to a homogeneous state characterized by a uniform density. These homogeneous fluid phases (*hom.fluid*) are distinguished from the liquid phase by the absence of a second peak in their radial distribution function (RDF) (Figure 4).

Well below the critical temperature, we observe coexistence phases with well-defined geometries of vapor or liquid phases, labeled *Coexis-Clear Morph* in Figure 3. At low densities (ρ* = 0.05 and 0.1), spherical droplets form. The intermediate density ρ* = 0.3 exhibits a cylindrical droplet, while higher densities (ρ* = 0.6 and 0.67) primarily show cylindrical bubbles. These configurations align with previous Monte Carlo simulations of LJ systems.^37^ This observation highlights the capability of HhPF to reproduce the formation of local configurations, as a function of the density of the system. As expected, nonperiodic spherical droplets are formed at low concentrations while cylinders, as well as other periodic morphologies, are formed only at higher densities.

Taking into account a range of densities beyond those currently evaluated, it is anticipated that a *slab* configuration will emerge at a density exceeding that of the cylindrical droplet configuration (0.3). Specifically, when the density is slightly greater than ρ* = 0.3, an increase in the radius of the cylinder will induce connections with its mirrored images, thereby forming slab configurations with a reduced surface-to-volume ratio. At a density higher than ρ* = 0.67, the vapor phase diminishes and fails to sustain a periodic cylindrical structure, leading predominantly to the observation of spherical bubble configurations.

The phases of coexistence seen in MGCM align with those observed in HhPF (Figure S2), with the exception of the case with ρ* = 0.6, which transforms into a slab. The densities that give rise to the slab configuration are known to be lower than those of the cylindrical bubble.^37^ This suggests that the effective density in HhPF is greater than in MGCM. We attribute this to the grid effect, which results in softer but effectively larger beads in the HhPF case.

Within the *Coexis-Clear Morph* region, increasing temperatures leads to a reduction in the size of predefined shapes (either spherical or cylindrical) and greater fluctuations at the interfaces. For example, at ρ* = 0.05, a rise in temperature causes the spherical droplet to shrink and adopt a more irregular form. Interestingly, at the highest simulated density of ρ* = 0.67, another microconfiguration (slab) appears at very low temperatures. This suggests that transitions between microconfigurations occur in the high-density coexistence phases. These transitions include changes in the interfacial geometry and present lower free energy barriers than the liquid–gas transition.

As temperatures near the critical point, the interface between the liquid and gas phases becomes indistinct, rendering the configurations less precise. These mixed phases are labeled *Coexist-Blur Morph* in Figure 3, due to the presence of a minor volume of the vapor phase. Starting from lower temperatures and moving into this area, we notice a reduction in droplet phases at low densities (ρ* = 0.05 and 0.1). In contrast, at higher densities (ρ* = 0.6 and 0.67), the vapor phase diminishes, resulting in the transition from cylindrical to spherical bubble configurations. As expected, the configurations in these domains are less distinct.

**Figure 5:**
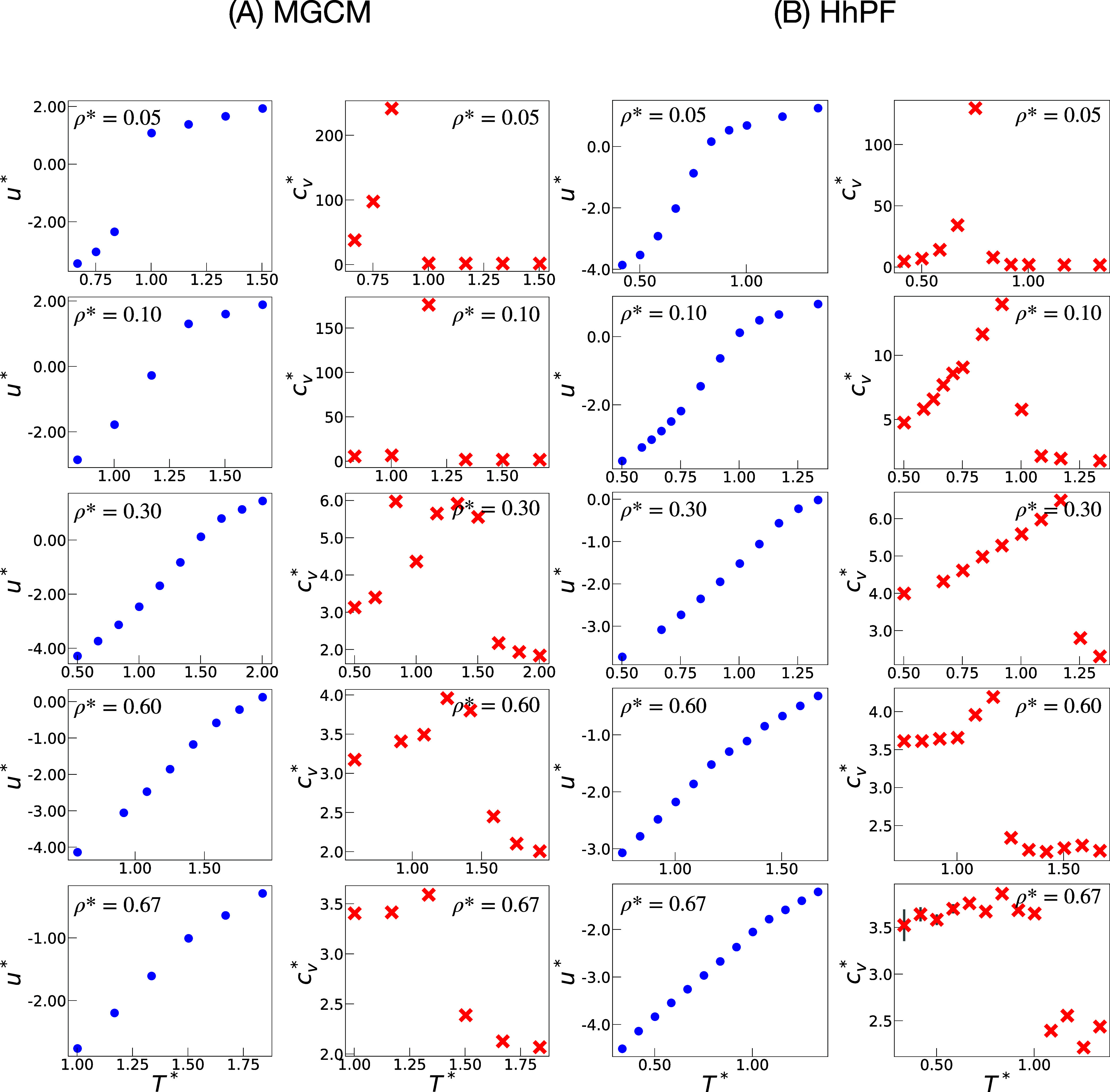
Variation of total energy per particle u* (blue/◦) and specific heat capacity  (red/×) at different densities ρ* with temperature T*. The results obtained from the MGCM simulations are shown in (A) and those from the HhPF simulations are shown in (B). All quantities are in reduced units.

The *phase categories* are supported by the internal energy and heat capacity curves in Figure 5. Transitions from homogeneous to coexistence phases show slope changes or discontinuities in internal energies and heat capacities. Homogeneous phases correspond to data points in internal energies with lower slopes, while coexistence phases have steeper slopes.

The difference in internal energy slope could be minor, but the heat capacity curve shows a noticeable discontinuity, dividing it into left and right groups. The right side represents homogeneous phases. The region referred to as *Coexis-Blur Morph* corresponds to the points (specifically, two points in our study) where there is a jump from the right side in the heat capacity values . The most significant example is the case of ρ* = 0.6, where the slope difference is small, but the homogeneous phases are clearly indicated by the six data points in the values of  on the right side.

The transition from a homogeneous phase to a coexisting phase is characterized as a droplet evaporation–condensation process, which is classified as a first-order phase transition.^37^ However, the morphological transition between distinct and indistinct phases of the coexisting state occurs more gradually and does not exhibit a pronounced discontinuity. Furthermore, the RDFs shown in Figure 4 assist in recognizing these phases. Homogeneous fluid phases at temperatures above the transition are characterized by the disappearance of the second peak in their distribution plots. When the liquid phase is present, a second peak emerges, and this peak becomes more prominent as the temperature decreases.

We can determine the phase transition boundaries according to the definition of these *phase categories*: either the entire transition zone or its upper limit, which lies between homogeneous fluids and the coexistence phase. We emphasize that the goal in this case is just broadly determining a region for the boundaries, not obtaining accurately the conditions of the phase transition. Upon comparing the transition regions of the HhPF and the MGCM results, it is noticeable that the phase boundary in the HhPF is moved to lower temperatures. This reduction in transition temperature is attributed to the shallower potential in HhPF, as previously discussed and illustrated in Figure 2. Note that the phase transition boundary for HhPF is more aligned with that of the LJ potential, both in shape and in critical temperatures. In contrast, the transition boundary for MGCM is significantly higher than that of LJ, because of the broader potential of MGCM, which leads to stronger interactions over longer distances following the first RDF peak.

At low densities, specifically ρ* = 0.05 and 0.1, variations in the transition boundary diminish substantially. This can be attributed to the larger spatial volume, where the significance of entropic contributions such as translational entropy becomes more dominant in their free energies, thereby reducing the impact of potential energy accordingly.

**Figure 6:**
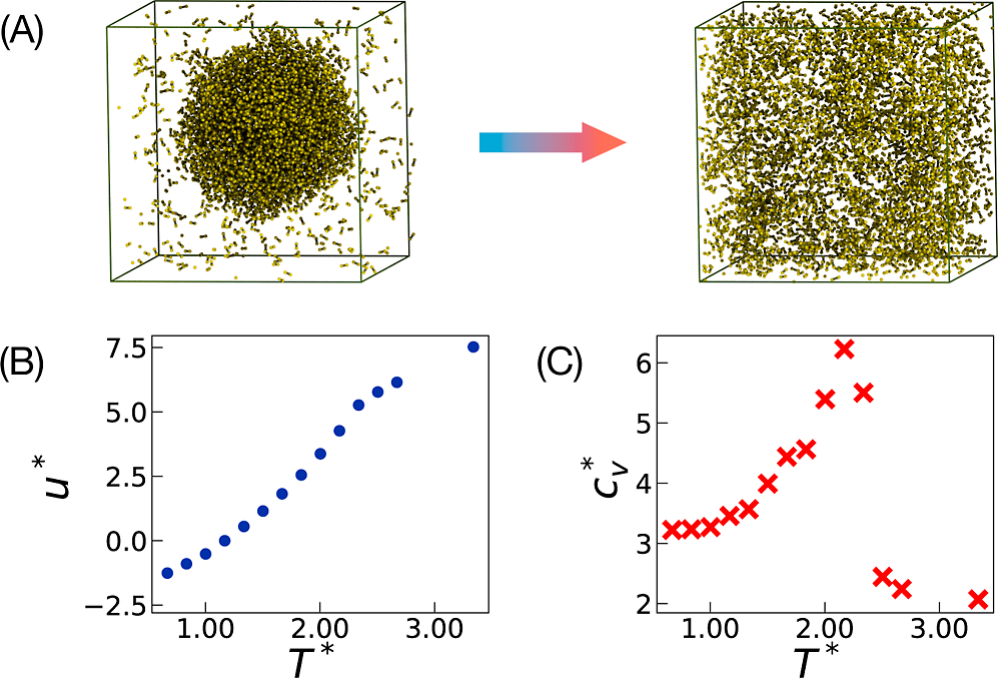
Results for the two-bead system simulation at density ρ* = 0.3. (A) Snapshots showing coexisting liquid–gas phases at temperature T* = 1.67 (left) and homogeneous fluid phase at T* = 2.67 (right). (B) Variation of total energy per particle u*, and (C) specific heat capacity  with temperature T*.

As a final test, we simulated a two-bead molecular system at ρ* = 0.3, where we observed a similar transition from liquid–gas coexistence to homogeneous fluid at temperatures higher than those for single-particle systems, as expected from the stronger intermolecular interactions. The variation of its internal energy and specific heat capacity with temperature is shown in Figure 6. Simulation details are provided in Supporting Information. This prototypical example shows how the same type of filter function can be used with more complex systems, including bonded terms, enabling the simulations of phase coexistence in such cases.

## Conclusion

The analysis presented in this study demonstrates the capability of the HhPF method with multi-Gaussian filters to accurately model liquid–gas coexistence across a wide range of thermodynamic conditions, capturing both macroscopic phase behavior and microscopic structural details. We have successfully integrated multi-Gaussian filters into the HhPF methodology, enhancing its capability to address phase coexistence phenomena. This advancement is analogous to the evolution from GCM to MGCM and has been implemented within the HyMD simulation package. By utilizing a linear combination of two Gaussian filters, we demonstrate that the HhPF approach can produce potentials with attractive components similar to LJ potentials, which are essential for modeling phase coexistence. This development extends the applicability of the hybrid particle-field methodology to phase coexistence studies without the need for modifications to the equation of state or the introduction of additional parameters.

Our implementation reveals that when filters are used to convey interaction shapes rather than merely smearing densities, the grid size must be smaller than half of the smallest characteristic distance, such as the width of the narrowest Gaussian filter. This requirement can increase computational costs, particularly when modeling strong repulsive interactions.

Simulations of systems across various densities and temperatures demonstrate that HhPF effectively captures detailed information on phase coexistence and interfacial phenomena through the use of multiple Gaussian filters. We observe microconfiguration transitions from low to high temperatures, increased interfacial fluctuations, and blurred interfaces in the transition area. The phase coexistence behaviors are consistent with both the MGCM and general LJ potentials. Interestingly, due to the broadening of potential fields caused by the grid, the phase boundary obtained from HhPF corresponds more closely to that of LJ systems, while the MGCM results show phase boundaries occurring at higher temperatures.

Our proposed framework allows for the utilization of any number of Gaussian functions in a linear combination to approximate complex potentials. Its ability to represent various phases, including coexistence, while maintaining the principles of HhPF, makes it a promising approach within mesoscale molecular simulation techniques. This advancement opens up new possibilities for studying complex systems and their phase behaviors at the mesoscopic level.

## Data Availability

HylleraasMD (HyMD), the HhPF code used to produce all the results contained in this work and the simulation data supporting the reported findings, is free and openly available in the GitHub repository https://github.com/Cascella-Group-UiO/HyMD. All simulation data supporting the findings reported here are openly available on the NIRD Research Data Archive at 10.11582/2024.00127.

## Supporting Information Available

Multi-Gaussian HhPF filter derivation, units conversion formulas, conditions and characteristics of simulated systems, dense mesh potential curves, phase diagram from MGCM simulations, and phase coexistence results for bonded two-bead systems (PDF)
