# Supplementary material for: Phase Coexistence in Hamiltonian Hybrid Particle–Field Theory Using a Multi-Gaussian Approach
Source: J Phys Chem B. 2024 Nov 14;128(47):11739–47. doi: 10.1021/acs.jpcb.4c05525 (PMC11613441; doi:10.1021/acs.jpcb.4c05525)
Supplement: Supplementary file 1 — jp4c05525_si_001.pdf [file jp4c05525_si_001.pdf]

Supporting Information for Phase coexistence in Hamiltonian hybrid particle–field theory using a Multi-Gaussian approach

Samiran Sen,[a] Henrique Musseli Cezar, Morten Ledum, Xinmeng Li,* and Michele Cascella*

Hylleraas Centre for Quantum Molecular Sciences and Department of Chemistry, University of Oslo, PO Box 1033 Blindern, 0315 Oslo, Norway

E-mail: xinmeng.li@kjemi.uio.no; michele.cascella@kjemi.uio.no

[a]Present address: Institut Pasteur, Université Paris Cité, CNRS UMR 3528, Computational Structural Biology Unit, F-75015 Paris, France

SI: Derivation of HhPF filter parameters

We consider a simple interaction energy of the form:

$$W^{H}_{MG}[\tilde{\phi}(\mathbf{r})] = \int d\mathbf{r}\, \tilde{\phi}(\mathbf{r})\tilde{\phi}(\mathbf{r}) \tag{1}$$

$$= \int d\mathbf{r} \sum_{i=1}^{N_G} g'_i(\mathbf{r})^2 + \int d\mathbf{r} \sum_{\substack{i,j=1\\ i\neq j}}^{N_G} g'_i(\mathbf{r}) g'_j(\mathbf{r}) \tag{2}$$

as already mentioned in the main text. While the first term can be straightforwardly evaluated to

$$\int d\mathbf{r}\, g'_i(\mathbf{r})^2 = \frac{a_i^2}{8\pi^{\frac{3}{2}}\sigma_{Hi}^3} \sum_{k=1}^{N}\sum_{l=1}^{N} \exp\left(-\frac{(\mathbf{r}_k-\mathbf{r}_l)^2}{4\sigma_{Hi}^2}\right) \quad , \tag{3}$$

carrying out the integral in the second term of (2) is more involved. We make use of the generalised Gaussian product-rule.[1] For each cross-term in the sum, we obtain:

$$\int d\mathbf{r}\, g'_i(\mathbf{r}) g'_j(\mathbf{r}) = \frac{a_i a_j}{(2\pi\sigma_{Hi}^2)^{\frac{3}{2}}(2\pi\sigma_{Hj}^2)^{\frac{3}{2}}} \int d\mathbf{r} \sum_{k=1}^{N}\sum_{l=1}^{N} \exp\left(-\frac{(\mathbf{r}-\mathbf{r}_k)^2}{2\sigma_{Hi}^2}\right) \exp\left(-\frac{(\mathbf{r}-\mathbf{r}_l)^2}{2\sigma_{Hj}^2}\right) \tag{4}$$

$$= \frac{a_i a_j}{(2\pi)^3\sigma_{Hi}^3\sigma_{Hj}^3} \sum_{k=1}^{N}\sum_{l=1}^{N} \exp\left(-\frac{(\mathbf{r}_k-\mathbf{r}_l)^2}{2(\sigma_{Hi}^2+\sigma_{Hj}^2)}\right) \times \int d\mathbf{r}\, \exp\left(-\frac{(\sigma_{Hi}^2+\sigma_{Hj}^2)}{2\sigma_{Hi}^2\sigma_{Hj}^2}(\mathbf{r}-\mathbf{P})^2\right) \tag{5}$$

where $\mathbf{P} = (\sigma_{Hj}^2\mathbf{r}_k + \sigma_{Hi}^2\mathbf{r}_l)/(\sigma_{Hj}^2+\sigma_{Hi}^2)$

The integral is now of standard form and gives:

$$\int d\mathbf{r}\, \exp\left(-\frac{(\sigma_{Hi}^2+\sigma_{Hj}^2)}{2\sigma_{Hi}^2\sigma_{Hj}^2}(\mathbf{r}-\mathbf{P})^2\right) = \left(\frac{2\pi\sigma_{Hi}^2\sigma_{Hj}^2}{(\sigma_{Hi}^2+\sigma_{Hj}^2)}\right)^{\frac{3}{2}} \tag{6}$$

which makes the cross-term:

$$\int d\mathbf{r}\, g_i'(\mathbf{r}) g_j'(\mathbf{r}) = \frac{a_i a_j}{(2\pi)^{\frac{3}{2}} \left(\sigma_{Hi}^2 + \sigma_{Hj}^2\right)^{\frac{3}{2}}} \sum_{k=1}^{N} \sum_{l=1}^{N} \exp\left(-\frac{(\mathbf{r}_k - \mathbf{r}_l)^2}{2(\sigma_{Hi}^2 + \sigma_{Hj}^2)}\right) \tag{7}$$

We can cast this cross-term in the form of the self-term as:

$$\int d\mathbf{r}\, g_i'(\mathbf{r}) g_j'(\mathbf{r}) = \frac{\alpha_{ij}^2}{8\pi^{\frac{3}{2}} \overline{\sigma}_{H_{ij}}^3} \sum_{k=1}^{N} \sum_{l=1}^{N} \exp\left(-\frac{(\mathbf{r}_k - \mathbf{r}_l)^2}{4\overline{\sigma}_{H_{ij}}^2}\right) \tag{8}$$

where,

$$\alpha_{ij} = \sqrt{a_i a_j} \tag{9}$$

$$\overline{\sigma}_{H_{ij}} = \sqrt{\frac{\sigma_{Hi}^2 + \sigma_{Hj}^2}{2}} \quad , \tag{10}$$

so that, both the self-term as well as the cross-term of the interaction energy functional (2) can be expressed in the same form. We note that α_{ij} is the geometric mean of a_i and a_j, and $\overline{\sigma}_{H_{ij}}^2$ is the arithmetic mean of σ_{Hi}^2 and σ_{Hi}^2.

SI: Reduced units

$$
\begin{aligned}
T^* &= \frac{T k_B}{\varepsilon_{LJ}} \\
\rho^* &= \rho \sigma_{LJ}^3 \\
r^* &= \frac{r}{\sigma_{LJ}}
\end{aligned}
\tag{11}
$$

where, $k_B = 8.314462618 \times 10^{-3}$ kJ mol^{-1} K^{-1} is the Boltzmann constant, $\sigma_{LJ} = 0.3405$ nm and $\varepsilon_{LJ} = 0.996$ kJ mol^{-1}.[2] $\rho = N/V$ is the particle number density, where $N = 8788$ is the total number of particles and V is the volume of the simulation box, T is the temperature and r is any distance.

For all simulations with monoatomic species, we explored reduced densities ρ^* from 0.05 to 0.67 and reduced temperatures T^* from 0.33 to 2.0.

SI: Conditions and characteristics of simulated systems

Table S1: Simulation conditions and observed phases and morphology for the HhPF simulations. a^* is the FCC lattice constant used to build the initial placement of the 8788 beads in our cubic simulation box. ρ^* and T^* are defined in the above section. All quantities are in reduced units.

a^*	ρ^*	T^*	Phase	Observed morphology
1.809	0.67	1.34	Single Gas	hom.vapor
1.809	0.67	1.25	Single Gas	hom.vapor
1.809	0.67	1.17	Single Gas	hom.vapor
1.809	0.67	1.09	Single Gas	hom.vapor
1.809	0.67	1.00	Coexis-Blur Morph	sph.bubble
1.809	0.67	0.92	Coexis-Blur Morph	cyl.bubble
1.809	0.67	0.84	Coexis-Clear Morph	cyl.bubble
1.809	0.67	0.75	Coexis-Clear Morph	cyl.bubble
1.809	0.67	0.67	Coexis-Clear Morph	cyl.bubble
1.809	0.67	0.58	Coexis-Clear Morph	cyl.bubble
1.809	0.67	0.50	Coexis-Clear Morph	slab
1.809	0.67	0.43	Coexis-Clear Morph	slab
1.809	0.67	0.33	Coexis-Clear Morph	slab
1.883	0.60	1.67	Single Gas	hom.vapor
1.883	0.60	1.59	Single Gas	hom.vapor
1.883	0.60	1.50	Single Gas	hom.vapor
1.883	0.60	1.42	Single Gas	hom.vapor
1.883	0.60	1.34	Single Gas	hom.vapor
1.883	0.60	1.25	Single Gas	hom.vapor
1.883	0.60	1.17	Coexis-Blur Morph	sph.bubble

Table S1: (continued)

1.883	0.60	1.09	Coexis-Blur Morph	cyl.bubble
1.883	0.60	1.00	Coexis-Clear Morph	cyl.bubble
1.883	0.60	0.92	Coexis-Clear Morph	cyl.bubble
1.883	0.60	0.84	Coexis-Clear Morph	cyl.bubble
1.883	0.60	0.75	Coexis-Clear Morph	cyl.bubble
2.370	0.30	1.34	Single Gas	hom.vapor
2.370	0.30	1.25	Single Gas	hom.vapor
2.370	0.30	1.17	Coexis-Blur Morph	hom.vapor
2.370	0.30	1.09	Coexis-Blur Morph	cyl.droplet
2.370	0.30	1.00	Coexis-Clear Morph	cyl.droplet
2.370	0.30	0.92	Coexis-Clear Morph	cyl.droplet
2.370	0.30	0.83	Coexis-Clear Morph	cyl.droplet
2.370	0.30	0.75	Coexis-Clear Morph	cyl.droplet
2.370	0.30	0.67	Coexis-Clear Morph	cyl.droplet
2.370	0.30	0.50	Coexis-Clear Morph	cyl.droplet
3.407	0.10	1.34	Single Gas	hom.vapor
3.407	0.10	1.17	Single Gas	hom.vapor
3.407	0.10	1.09	Single Gas	hom.vapor
3.407	0.10	1.00	Coexis-Blur Morph	sph.droplet
3.407	0.10	0.92	Coexis-Blur Morph	sph.droplet
3.407	0.10	0.83	Coexis-Clear Morph	sph.droplet
3.407	0.10	0.75	Coexis-Clear Morph	sph.droplet
3.407	0.10	0.71	Coexis-Clear Morph	sph.droplet
3.407	0.10	0.67	Coexis-Clear Morph	sph.droplet
3.407	0.10	0.63	Coexis-Clear Morph	sph.droplet
3.407	0.10	0.58	Coexis-Clear Morph	sph.droplet

Table S1: (continued)

3.407	0.10	0.50	Coexis-Clear Morph	sph.droplet
4.308	0.05	1.34	Single Gas	hom.vapor
4.308	0.05	1.17	Single Gas	hom.vapor
4.308	0.05	1.00	Single Gas	hom.vapor
4.308	0.05	0.92	Single Gas	hom.vapor
4.308	0.05	0.83	Coexis-Blur Morph	sph.droplet
4.308	0.05	0.75	Coexis-Clear Morph	sph.droplet
4.308	0.05	0.67	Coexis-Clear Morph	sph.droplet
4.308	0.05	0.58	Coexis-Clear Morph	sph.droplet
4.308	0.05	0.50	Coexis-Clear Morph	sph.droplet
4.308	0.05	0.42	Coexis-Clear Morph	sph.droplet

Table S2: Simulation conditions and observed phases and morphology for the MGCM simulations. a^* is the FCC lattice constant used to build the initial placement of the 8788 beads in our cubic simulation box. ρ^* and T^* are defined in the above section. All quantities are in reduced units.

a^*	ρ^*	T^*	Phase	Observed morphology
1.809	0.67	1.84	Single Gas	hom.vapor
1.809	0.67	1.67	Single Gas	hom.vapor
1.809	0.67	1.50	Single Gas	hom.vapor
1.809	0.67	1.34	Coexis-Blur Morph	cyl.bubble
1.809	0.67	1.17	Coexis-Clear Morph	cyl.bubble
1.809	0.67	1.00	Coexis-Clear Morph	cyl.bubble
1.883	0.60	1.92	Single Gas	hom.vapor
1.883	0.60	1.75	Single Gas	hom.vapor
1.883	0.60	1.59	Single Gas	hom.vapor
1.883	0.60	1.42	Coexis-Blur Morph	sph.bubble
1.883	0.60	1.25	Coexis-Clear Morph	slab
1.883	0.60	1.09	Coexis-Clear Morph	slab
1.883	0.60	0.92	Coexis-Clear Morph	slab
1.883	0.60	0.58	Coexis-Clear Morph	slab
2.370	0.30	2.00	Single Gas	hom.vapor
2.370	0.30	1.84	Single Gas	hom.vapor
2.370	0.30	1.67	Single Gas	hom.vapor
2.370	0.30	1.50	Coexis-Blur Morph	sph.droplet
2.370	0.30	1.34	Coexis-Clear Morph	cyl.droplet
2.370	0.30	1.17	Coexis-Clear Morph	cyl.droplet
2.370	0.30	1.00	Coexis-Clear Morph	cyl.droplet

Table S2: (continued)

2.370	0.30	0.84	Coexis-Clear Morph	cyl.droplet
2.370	0.30	0.67	Coexis-Clear Morph	cyl.droplet
2.370	0.30	0.50	Coexis-Clear Morph	cyl.droplet
3.407	0.10	1.67	Single Gas	hom.vapor
3.407	0.10	1.50	Single Gas	hom.vapor
3.407	0.10	1.34	Single Gas	hom.vapor
3.407	0.10	1.17	Coexis-Blur Morph	sph.droplet
3.407	0.10	1.00	Coexis-Clear Morph	sph.droplet
3.407	0.10	0.84	Coexis-Clear Morph	sph.droplet
4.308	0.05	1.50	Single Gas	hom.vapor
4.308	0.05	1.34	Single Gas	hom.vapor
4.308	0.05	1.17	Single Gas	hom.vapor
4.308	0.05	1.00	Single Gas	hom.vapor
4.308	0.05	0.84	Coexis-Clear Morph	sph.droplet
4.308	0.05	0.75	Coexis-Clear Morph	sph.droplet
4.308	0.05	0.67	Coexis-Clear Morph	sph.droplet
4.308	0.05	0.50	Coexis-Clear Morph	sph.droplet
4.308	0.05	0.42	Coexis-Clear Morph	sph.droplet

SI: Dense mesh potential curves

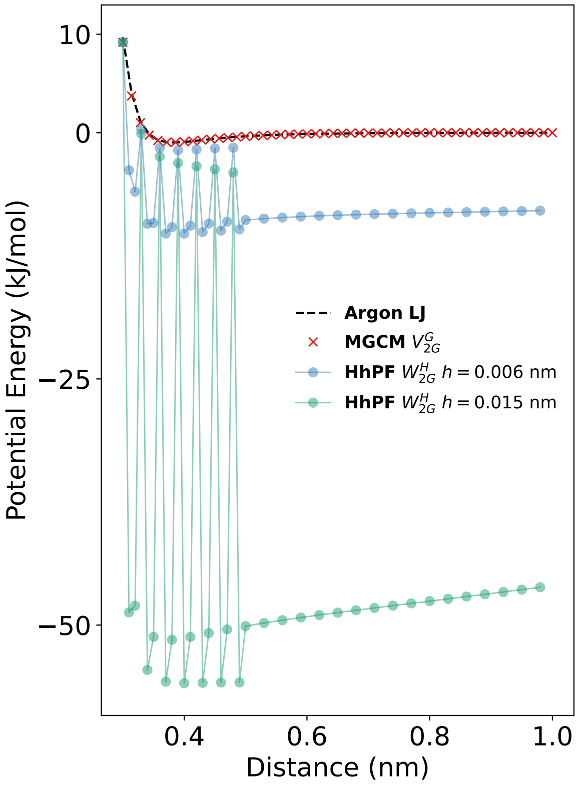

Figure S1: A two-Gaussian potential V_{2G}^{G} capable of replicating the Argon LJ potential. The potential energy W_{2G}^{H} is obtained from HhPF simulations of two beads for grid resolutions $h = 0.006$ and 0.016 nm. The Gaussian parameters for V_{2G}^{G} are a_{G1} = -14592.966, a_{G2} = 5.078, σ_{G1} = 0.112, and σ_{G2} = 0.310. In W_{2G}^{H}, the filter parameters are a_{H1} = 10.736, a_{H2} = -0.039, σ_{H1} = 0.056, and σ_{H2} = 0.212. The unit for the weight parameter a is kJ/mol, and for width parameter σ is nm.

SI: Phase diagram from MGCM and HhPF simulations

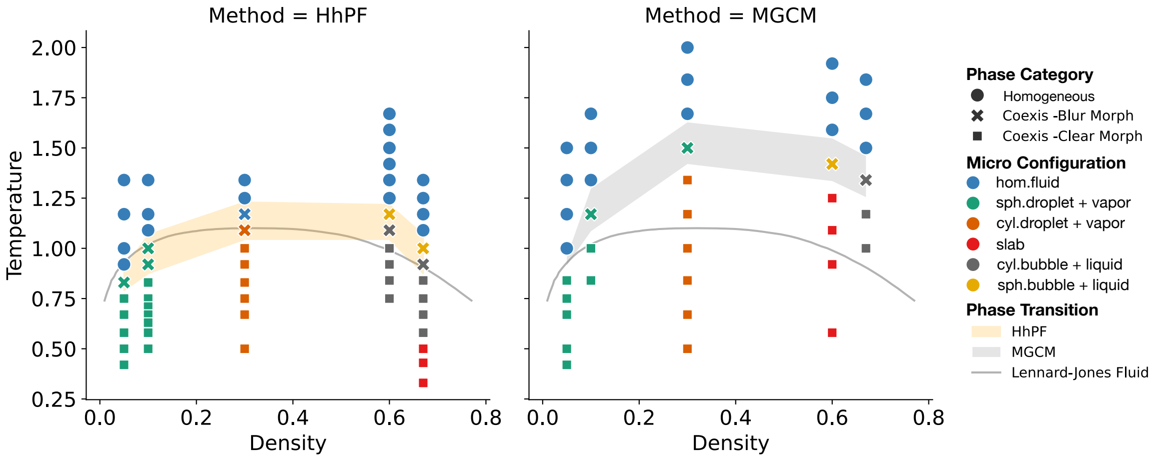

Figure S2: *(Right)* Phase diagram results of MGCM simulations. *(Left)* Results of HhPF simulations (as shown in Figure 3 of the main text), reproduced for comparison.

SI: Bonded two-bead system

HhPF simulations of a bonded two-bead system comprising 4394 molecules were performed at a density of $\rho^* = 0.3$ in a temperature range of $T^* = 0.67$ to 3.34 with bond-length 0.153 nm and bond-strength 1000 kJ mol^{-1}. Simulations were run for 50ns with a time-step of 0.1ps and grid-spacing of 0.06nm. All the other simulation parameters are the same as in the monoatomic simulations.

References

(S1) Boys, S. F. Electronic wave functions-I. A general method of calculation for the stationary states of any molecular system. *P. Roy. Soc. Lond. A Mat.* **1950**, *200*, 542–554.

(S2) Lin, S.-T.; Blanco, M.; Goddard, W. A. The two-phase model for calculating thermodynamic properties of liquids from molecular dynamics: Validation for the phase diagram of Lennard-Jones fluids. *J. Chem. Phys* **2003**, *119*, 11792–11805.
